# Construction of a Novel Prognostic Signature in Lung Adenocarcinoma Based on Necroptosis-Related lncRNAs

**DOI:** 10.3389/fgene.2022.833362

**Published:** 2022-07-22

**Authors:** Xiayao Diao, Chao Guo, Shanqing Li

**Affiliations:** Department of Thoracic Surgery, Peking Union Medical College Hospital, Chinese Academy of Medical Sciences and Peking Union Medical College, Beijing, China

**Keywords:** necroptosis, long non-coding RNA, prognostic signature, lung adenocarcinoma, overall survival

## Abstract

**Background:** Long non-coding RNAs (lncRNAs) are drawing increasing attention as promising predictors of prognosis for lung adenocarcinoma (LUAD) patients. Necroptosis, a novel regulated mechanism of necrotic cell death, plays an important role in the biological process of cancer. The aim of this study was to identify the necroptosis-related lncRNAs (NRLRs) in a LUAD cohort and establish a necroptosis-related lncRNA signature (NRLSig) to stratify LUAD patients.

**Methods:** NRLRs were identified in LUAD patients from The Cancer Genome Atlas (TCGA) database using Pearson correlation analysis between necroptosis-related genes and lncRNAs. Then the NRLSig was identified using univariate Cox regression analysis and LASSO regression analysis. Assessments of the signature were performed based on survival analysis, receiver operating characteristic (ROC) curve analysis and clustering analysis. Next, a nomogram containing the NRLSig and clinical information was developed through univariate and multivariate Cox regression analysis. Further, functional enrichment analysis of the selected lncRNAs in NRLSig and the association between NRLSig and the immune infiltration were also evaluated.

**Results:** A 4-lncRNA signature, incorporating LINC00941, AP001453.2, AC026368.1, and AC236972.3, was identified to predict overall survival (OS) and stratify LUAD patients into different groups. Survival analysis, ROC curve analysis and clustering analysis showed good performance in the prognostic prediction of the lncRNA signature. Then, a nomogram containing the NRLSig was developed and showed satisfactory predictive accuracy, calibration and clinical usefulness. The co-expressed genes of selected NRLRs were enriched in several biological functions and signaling pathways. Finally, differences in the abundance of immune cells were investigated among the high-risk group and low-risk group divided by the NRLSig.

**Conclusion:** The proposed NRLSig may provide promising therapeutic targets or prognostic predictors for LUAD patients.

## Introduction

Lung cancer is one of the most common malignancies and the leading cause of cancer-associated deaths worldwide (Siegel et al., 2020). Non-small cell lung cancer (NSCLC) is the most frequently reported subtype, accounting for approximately 85% of all lung cancer cases (Meza et al., 2015). NSCLC includes three main histological subtypes: adenocarcinoma, squamous cell carcinoma and large cell carcinoma (Zappa and Mousa, 2016). Among them, Lung adenocarcinoma (LUAD) is the most prevalent histotype. Although diagnostic techniques and therapeutic strategies have been developed for LUAD patients, the 5-year overall survival (OS) rate of them remains only 15% (Miller et al., 2012). Therefore, it is urgent to identify some novel effective diagnostic markers, therapeutic targets, and prognostic factors to offer early diagnosis, timely treatment, and precise prediction for LUAD patients.

Tumorigenesis and drug resistance are often attribute to resistance to apoptosis in most tumors (Johnstone et al., 2002; Pan et al., 2021). This phenomenon calls for identifying strategies to induce non-apoptotic approaches of programmed cell death as promising novel therapeutics in cancer (Tang R. et al., 2020). Necrosis used to be recognized as a completely opposite form of cell death compared to apoptosis (Linkermann and Green, 2014). However, necroptosis, an alternative regulated cell death, can be elicited by the activation of various signaling pathways, tumor microenvironmental stresses, or multiple chemotherapeutic drugs (Huang et al., 2013; Lalaoui et al., 2015; Galluzzi et al., 2018). Emerging evidence illustrates that necroptosis act as a crucial approach in the regulation of biological processes of tumor, including oncogenesis, progression, metastasis, cancer immunity, and cancer subtypes (Stoll et al., 2017; Seehawer et al., 2018). Manipulating or targeting the necroptotic pathway may also play an important role for bypassing resistance of apoptosis, supporting anti-cancer immunity in cancer therapy and predicting prognosis for cancer patients (Gong et al., 2019).

Long non-coding RNAs (lncRNAs), non-protein-coding transcripts longer than 200 ribonucleotides, play a pivotal role in gene regulation (Agostini et al., 2020). LncRNAs participate in various biological processes, such as immune, metabolism, infection, and more (Gibb et al., 2011; Tan et al., 2021). In addition, lncRNAs exert these functions by interacting with other molecules such as RNA, DNA, and proteins (Md Yusof et al., 2020). In recent years, with the development of high-throughput sequencing techniques, increasing studies have demonstrated many non-coding genes play an important role in the development and progression of tumors. LncRNAs have also been revealed to function as regulators in cancer biology, including proliferation, invasion, and metastasis (Hung et al., 2014; Kim et al., 2014), as well as tumor angiogenesis or lymphangiogenesis (Prensner et al., 2014; He et al., 2018). Notably, it has been revealed that several lncRNAs may act as mediators regulating necroptosis in different tumors. For example, lncRNA H19, as a precursor of miR-675, regulates necroptosis *via* miR-675 in hepatocellular carcinoma (Harari-Steinfeld et al., 2021). Moreover, 16 lncRNAs associated with necroptosis were also identified in gastric cancer patients through bioinformatic analysis (Zhao et al., 2021). However, only small amount of lncRNAs, especially the NRLRs, have been functionally or prognostically well-characterized. Therefore, it is valuable to identify key lncRNAs closely related to necroptosis with prognosis significance in LUAD.

In present study, the lncRNAs expression profiles of LUAD patients were collected from public database. We then developed a necroptosis-related lncRNA signature (NRLSig) and systematically evaluated the associations of necroptosis-related lncRNAs (NRLRs) with the prognosis and clinical or pathological characteristics of LUAD patients. Moreover, we established a nomogram that incorporates the NRLSig and clinical factors to further stratify these patients. The high-risk group and low-risk group identified by NRLSig were compared based on various factors, including tumor-infiltrating immune cells and principal component analysis (PCA). Finally, functional enrichment analysis was also conducted to explore the potential mechanism of the selected lncRNAs. This study revealed the prognostic value of NRLRs in LUAD and constructed a prognostic model to evaluate prognosis of LUAD patients.

## Materials and Methods

### Data Collection

The RNA transcriptome datasets of 535 LUAD patients, including 535 tumor samples and 59 adjacent normal samples, were obtained from The Cancer Genome Atlas (TCGA) (https://portal.gdc.cancer.gov). The detailed clinicopathological information, including survival status, survival time, age, gender, TNM stage, T stage, N stage, and M stage, were also downloaded from the above dataset. Only LUAD patients with clear survival time and survival status were included in the study. Patients whose OS was less than 30 days in the TCGA-LUAD database were excluded to reduce statistical bias in this analysis. With corresponding clinical information, the LUAD patients who fit the criteria above were divided into a training set and validation set randomly in a 1:1 ratio by using the “caret” R package.

### Identification of Necroptosis-Related lncRNAs in Lung Adenocarcinoma

According to the lncRNAs annotation file obtained from the GENCODE (https://www.gencodegenes.org/) (Derrien et al., 2012), 14,128 lncRNAs were acquired from the RNA transcriptome datasets of the TCGA-LUAD. The differentially expressed lncRNAs between LUAD and normal tissues were identified in the TCGA cohort with false discovery rate (FDR) < 0.05 and |Log_2_ fold change (FC)| ≥ 1 (Glickman et al., 2014). The differential expression analysis was conducted using the “limma” package. To visualize the screening results for differentially expressed lncRNAs, we also plotted the heatmap and volcano plot using the “pheatmap” R package. Moreover, necroptosis-related genes (NRGs) were identified from two sources. 159 NRGs were extracted in the necroptosis pathway (hsa04217) from the KEGG PATHWAY database (https://www.kegg.jp/). 67 NRGs were obtained from literature research (Zhao et al., 2021). Finally, a total of 204 NRGs were included for subsequent research after integrating intersection from these two gene sets (Supplementary Table S1). Pearson correlation analysis was conducted between the differentially expressed lncRNAs and 204 NRGs (with the |Correlation Coefficient| > 0.3 and *p* < 0.001) to identify NRLRs using the “limma” package.

### Establishment of the Prognostic Necroptosis-Related lncRNA Signature for Lung Adenocarcinoma

According to the corresponding survival information of LUAD cases in the training set, univariate Cox analysis for association with OS was conducted to identify the prognostic NRLRs. Finally, the lncRNAs with *p* value < 0.05 were selected to further establish the NRLSig through the least absolute shrinkage and selection operator (LASSO) regression algorithm using the “glmnet” package in R software (Tibshirani, 1997; Simon et al., 2011), and 10-fold cross-validation was utilized to filtrate candidate NRLRs and identify the penalty parameter (λ), corresponding to the minimum value of partial likelihood deviance. A risk signature was then developed based on the risk coefficients and the expression levels of optimal prognostic lncRNAs. The prognostic risk score formula was constructed as follows:

$$Risk\;score=\sum_{i=1}^{n}coefficients*Expression\;of\;NRLRs\left(i\right)$$

### Assessment of the Prognostic Signature Incorporating Necroptosis-Related lncRNAs

LUAD patients in the training set were identified into high-risk group and low-risk group according to the median value of the risk score. Kaplan–Meier survival analysis was performed to evaluate the survival difference between these two groups using the “survival” and “survminer” R packages. The discrimination performance of the NRLSig was also evaluated through the time-dependent receiver operating characteristic (ROC) curve analysis using “timeROC” R package. The consistent formula and cutoff point (the median of risk scores in the training set) were also used to calculate the risk score of each patient in internal validation set and divided into high-risk group and low-risk group. Then, survival analyses and ROC curve analyses were conducted in the validation set and the entire TCGA-LUAD dataset. In addition, principal component analysis (PCA) was performed using “limma” and “scatterplot3d” packages to estimate the clustering ability of prognostic signature. Besides, Kaplan–Meier survival analysis was conducted to examine prognostic significance in each subgroup categorized by clinicopathological features.

### Development and Assessment of the Nomogram Containing Necroptosis-Related lncRNA Signature

We further identified whether the risk score generated from the NRLSig and clinicopathological predictors, including age, gender, TNM stage, T stage, N stage, and M stage, were independent prognostic predictors of OS in the entire set through univariate and multivariate Cox regression analysis. Then, we formulated a nomogram based on identified independent variable factors using the “rms” R package. Moreover, the prognostic value of the nomogram was evaluated by the Kaplan–Meier survival analysis based on the high-risk group and low-risk group divided by the median value of the risk score, generated from the nomogram. The discrimination and calibration of the nomogram was estimated in the entire TCGA-LUAD dataset by the ROC curves and calibration curves. Besides, the decision curve analysis (DCA) was utilized to evaluate the clinical usefulness of the model through calculating the net benefits at different threshold probabilities.

### Functional Enrichment Analysis and Immune Cell Characteristic Analysis

To explore the biological functions of the selected lncRNAs in NRLSig, we identified the protein-coding genes significantly associated with these lncRNAs from the TCGA dataset through co-expression network analysis. The |Pearson correlation coefficients| > 0.5 and *p* < 0.001 were considered as criteria for significantly correlation. We further performed Gene Ontology (GO) and Kyoto Encyclopedia of Genes and Genomes (KEGG) enrichment analyses of the NRGs to investigate the functions of the genes selected above. The functions or pathways with *p*-Value < 0.05 were regarded as significantly enriched. Functional enrichment analysis was performed using the “clusterProfiler” R package.

The CIBERSORT (Newman et al., 2015; Chen et al., 2018) and TIMER (Li J. et al., 2020; Li T. et al., 2020) algorithms were utilized to analyse the abundances of tumor-infiltrating immune cells among the each LUAD patients in the TCGA dataset. Moreover, the abundances for 22 types of immune cells of the patients in the high-risk group and low-risk group stratified by the NRLSig, including naive CD4^+^ T cells, resting memory CD4^+^ T cells, activated memory CD4^+^ T cells, naive B cells, memory B cells, plasma cells, CD8^+^ T cells, follicular helper T cells, regulatory T cells, gamma delta T cells, M0 macrophages, M1 macrophages, M2 macrophages, resting natural killer cells, activated natural killer cells, monocytes, resting dendritic cells, activated dendritic cells, resting mast cells, activated mast cells, eosinophils, and neutrophils, were compared and visualized using the CIBERSORT algorithm. In addition, the association between the NRLSig and immune infiltration cells, including B cells, CD4^+^ T cells, CD8^+^ T cells, dendritic cells, macrophages, and neutrophils, were also analyzed using the TIMER algorithm.

### Tissue Sample Collection and Lung Adenocarcinoma Cell Culture

A total of 12 pairs of LUAD tissues and noncancerous adjacent tissues (NAT) were collected from patients who had undergone surgical resection at the Department of Thoracic Surgery, Peking Union Medical College Hospital (Beijing, China). Written informed consent was obtained from all patients before collection. This study was approved by the Institutional Ethics Review Committee at Peking Union Medical College Hospital and was conducted in accordance with recognized ethical guidelines. All samples were stored at −80°C.

All human LUAD cell lines (A549, H1299, and PC9) and human bronchial epithelial cell line (BEAS-2B) were purchased from the American Type Culture Collection (ATCC). A549 and BEAS-2B cells were cultured in DMEM medium (Gibco). H1299 and PC9 cells were cultured in RPMI 1640 medium (Gibco). All medium was supplemented with 10% fetal bovine serum (BI). All cells were maintained in a humidified incubator with 5% CO_2_ at 37°C.

### RNA Extraction and qRT-PCR Analysis

Total cellular and tissue RNA was extracted using Trizol reagent (Takara Bio, Japan) following the manufacturer’s protocols. Then, RNA samples were reverse transcribed by Hiscript III Reverse Transcriptase kit (Vazyme, Nanjing, China) and corresponding RNA expression was evaluated by qRT-PCR with ChamQ^TM^ Universal SYBR qPCR Master Mix kit (Vazyme). GAPDH acted as the internal reference for normalization. The detailed sequence of primers used were listed in Supplementary Table S2.

### Statistical Analysis

All statistical analyses were conducted using the R software, version 4.0.2 (https://www.r-project.org). Pearson correlation analysis was used to analyze the correlation between NRGs and NRLRs. Differences in the proportions of clinical characteristics, such as age, gender, and T stage, were analyzed by the chi-squared test. The Mann-Whitney *U* test was implemented to compare the expression of genes or lncRNA, and abundance of tumor-infiltrating immune cells. Univariate Cox regression analysis and LASSO regression analysis or multivariate Cox regression were conducted to define the optimal prognostic factor for OS. The OS between high-risk group and low-risk group was compared using the Kaplan-Meier analysis with the log-rank test. All statistical tests were two-tailed, and *p* < 0.05 was considered statistically significant.

## Results

### Identification of Necroptosis-Related lncRNAs in Lung Adenocarcinoma Patients

**FIGURE 1:**
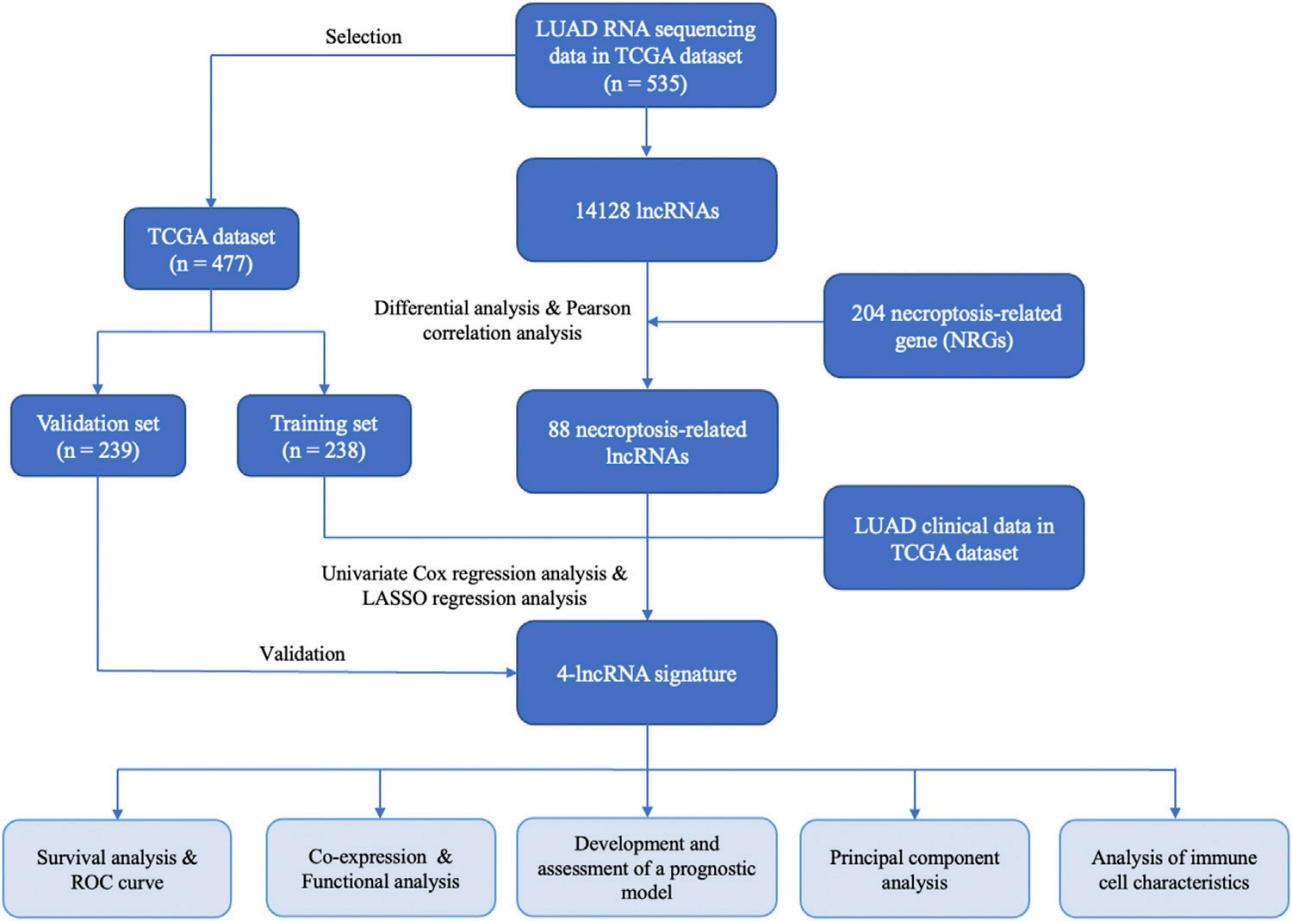
The flow chart of key steps in this study.

**FIGURE 2:**
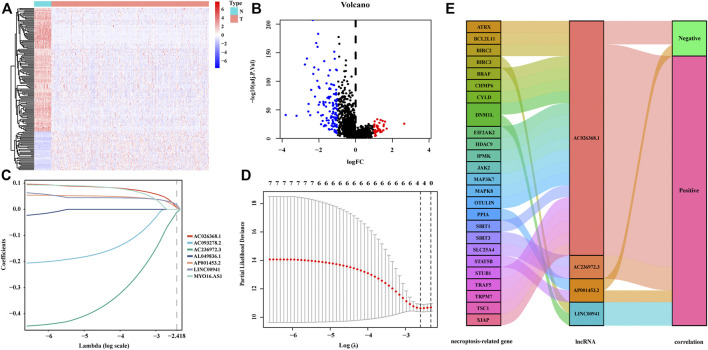
Construction of the signature incorporating necroptosis-related lncRNAs. The heatmap **(A)** and the volcano plot **(B)** of differentially expressed lncRNAs in LUAD patients. **(C,D)** The LASSO coefficient profiles of the 7 lncRNAs at different tuning parameters (λ), 10-fold cross-validation to filtrate candidate necroptosis-related lncRNAs in LASSO regression analysis. **(E)** The Sankey diagram of the correlation between necroptosis-related genes (NRGs) and lncRNAs. The left bar represents the NRGs. The median bar represents the selected lncRNAs. The right bar represents the correlations between NRGs and lncRNAs generated from the Pearson correlation analysis.

The flow chart for the risk signature development and subsequent analyses is illustrated in Figure 1. A total of 535 LUAD patients with RNA sequencing data were included in present study. Among 14,128 lncRNAs identified, 696 differentially expressed lncRNAs were significant between tumor and adjacent normal tissues (Figures 2A,B). According to these lncRNAs and 204 NRGs, NRLRs were identified through Pearson correlation analysis (|Correlation Coefficient| > 0.3 and *p* < 0.001). Finally, 88 NRLRs were selected for subsequent analyses (Supplementary Figure S1).

### Construction of the Prognostic Necroptosis-Related lncRNA Signature for Lung Adenocarcinoma Patients

To develop the NRLSig for predicting the survival status of LUAD patients, a total of 477 patients, who meet the inclusion and exclusion criteria, were randomly grouped into a training set (238 patients) and a validation set (239 patients) in a 1:1 ratio. The baseline characteristics of the entire TCGA-LUAD patients are summarized in Table 1. Based on the transcription profile of NRLRs, 7 NRLRs were found associated with the OS of LUAD patients in univariate Cox proportional hazards regression analysis. The lncRNAs with *p*-Value < 0.05 were selected for LASSO regression analysis to further identify optimal prognostic lncRNAs. Finally, a 4-lncRNA signature was constructed based on the optimal value of λ (Figures 2C,D). According to the coefficient values, the formula of the NRLsig was presented as follows: risk score=(0.0286×LINC00941)+(0.0226×AP001453.2)+(0.0328×AC026368.1)+(−0.0499×AC236972.3) . Besides, in the Sankey diagram, we identified 22 NRGs were positively correlated to the selected NRLRs, while STAT5B, CHMP6 and STUB1 were negatively associated with the lncRNAs (Figure 2E).

**TABLE 1:** Clinical features of selected lung adenocarcinoma (LUAD) patients in TCGA dataset. TCGA, The Cancer Genome Atlas.

Characteristic N (477) %	N (477)	%
Gender		
Male	220	46.1
Female	257	53.9
Age, years		
≤65	230	48.2
>65	247	51.8
TNM stage		
Stage I	253	53.0
Stage II	113	23.7
Stage III	78	16.4
Stage IV	25	5.2
Unknown	8	1.7
T stage		
T_1_	159	33.3
T_2_	254	53.2
T_3_	43	9.0
T_4_	18	3.8
Unknown	3	0.6
N stage		
N_0_	307	64.4
N_1_	90	18.9
N_2_	67	14.0
N_3_	2	0.4
Unknown	11	2.3
M stage		
M_0_	447	93,7
M_1_	26	5.5
Unknown	4	0.8
Survival status		
Alive	320	67.1
Dead	157	32.8

**FIGURE 3:**
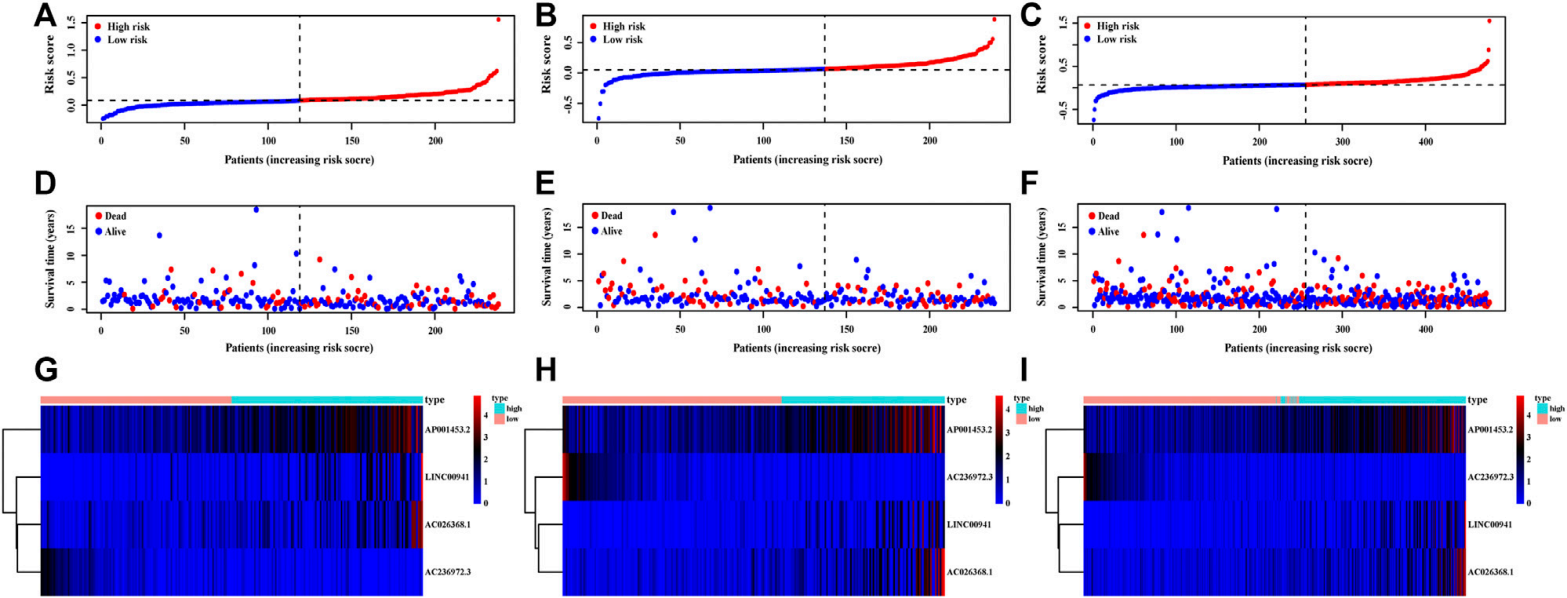
Distribution of LUAD patients stratified by the necroptosis-related lncRNA signature (NRLsig). **(A–C)** The distribution of low-risk group and high-risk group LUAD patients divided by the NRLsig in the training, validation, and entire set, respectively. **(D–F)** Survival statuses of patients in different groups stratified by the NRLsig in the training, validation, and entire set, respectively. **(G–I)** Heatmap of expression statuses of the selected necroptosis-related lncRNAs in the training, validation, and entire set, respectively.

**FIGURE 4:**
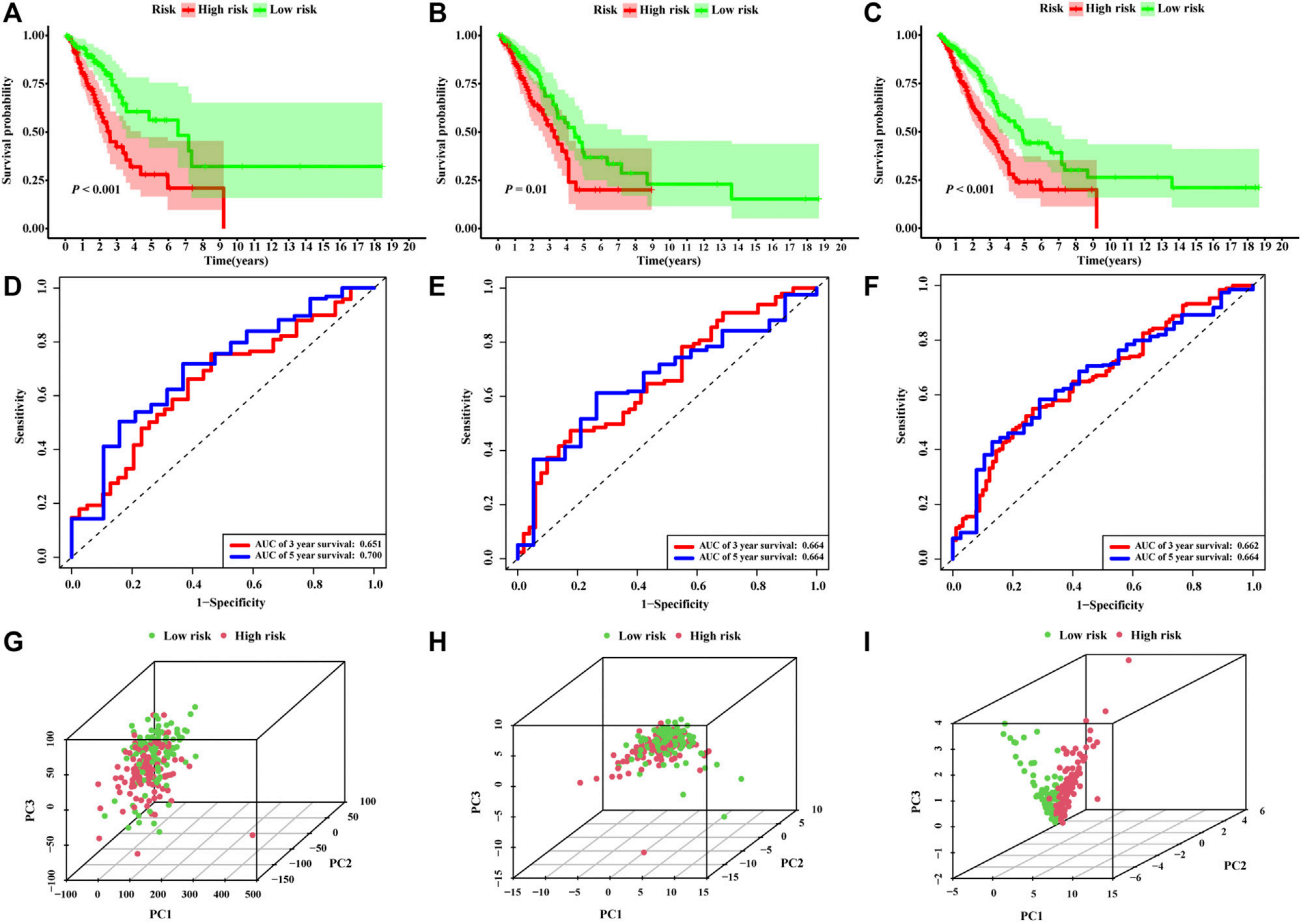
Assessments of the necroptosis-related lncRNA signature (NRLsig). **(A–C)** Kaplan–Meier survival analysis curves of the high-risk group and low-risk group stratified by NRLsig in the training, validation, and entire TCGA-LUAD set, respectively. **(D–F)** Time-dependent ROC curves at 3- and 5-year follow-up in the training, validation and entire TCGA-LUAD set, respectively. Principal component analysis (PCA) of the low-risk group and high-risk group stratified by the whole-genome **(G)**, necroptosis-related genes **(H)**, and the NRLsig **(I)**.

The risk score of each patient was calculated based on the formula in the training set, validation set, and entire set, and the median of risk scores, as the determined cutoff value, were used to classify patients into a low-risk group or high-risk group (Figures 3A–C). The distribution of survival status in each set was plotted in Figures 3D–F. These figures illustrated that increasing risk score was positively associated with accumulating number of patients with poor prognoses. The expression levels of the lncRNAs selected in the signature were also showed in Figures 3G–I. Survival analyses illustrated that the patients in high-risk group possessed significantly lower survival rate compared to patients in the low-risk group in all three sets (*p* < 0.001, *p* = 0.01 and *p* < 0.001, respectively, Figures 4A–C). The AUC of the NRLSig at 3- and 5-year also showed a good discriminative performance in the training set, validation set, and entire set (Figures 4D–F). As depicted in Figures 4G,H, the high-risk group and low-risk group could not be effectively identified using the whole genome or necroptosis-related genes; however, LUAD patients could be clearly classified into high-risk or low-risk group using NRLSig (Figure 4I), further supporting the performance of the lncRNA signature. Survival analysis in subgroups was also conducted and showed significant differences in prognosis among the low-risk group and high-risk group, except for TNM stage III-IV and M1 stage patients, which suggested that the prognostic signature was applicable to different subtypes of LUAD patients (Supplementary Figures S2A–L). All these assessments indicated that NRLSig is a reliable independent prognostic risk factor for patients with LUAD.

### Development and Performance Assessment of the Nomogram Incorporating the Necroptosis-Related lncRNA Signature

**FIGURE 5:**
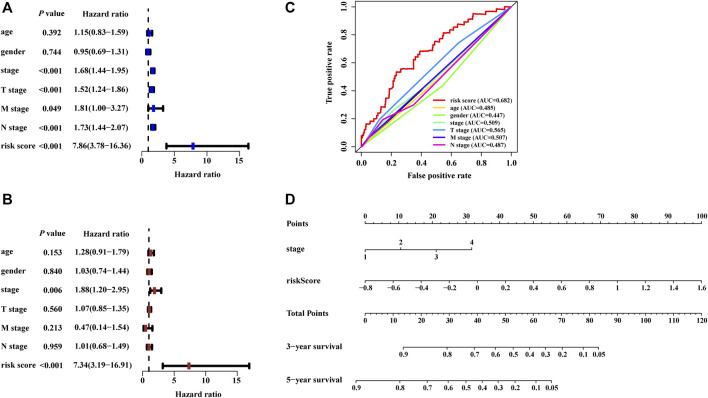
Development of the nomogram based on the risk scores calculated from the signature. **(A,B)** The predictors extracted by univariate and multivariate Cox analyses. **(C)** Time-dependent ROC curves for predicting overall survival at 1 year by risk score, age, gender, stage, T stage, M stage, and N stage. **(D)** A prognostic nomogram was developed to predict the 3- and 5-year survival for LUAD patients.

The risk score calculated from the NRLSig and several clinical candidate factors were evaluated by the univariate and multivariate Cox regression algorithm in the entire LUAD set. Univariate Cox regression analysis revealed that the risk score of the signature was correlated with the OS of LUAD patients (*p* < 0.001, Figure 5A). Multivariate Cox regression analysis further demonstrated that the risk signature was an independent prognostic factor for predicting the OS of LUAD patients (*p* < 0.001, Figure 5B). We also performed time-dependent ROC curves of 1-year OS, and the AUC value for the risk score generated from the NRLSig was 0.682, which was higher than other clinical predictors, further supporting the discriminative power of NRLSig for predicting survival status in LUAD (Figure 5C). All variables which were significant (*p* < 0.05) in the multivariate Cox regression analysis were included in the predictive model. Finally, a nomogram to predict the 3- and 5-year OS was constructed incorporating the risk score generated from the NRLSig and the TNM stage (Figure 5D).

**FIGURE 6:**
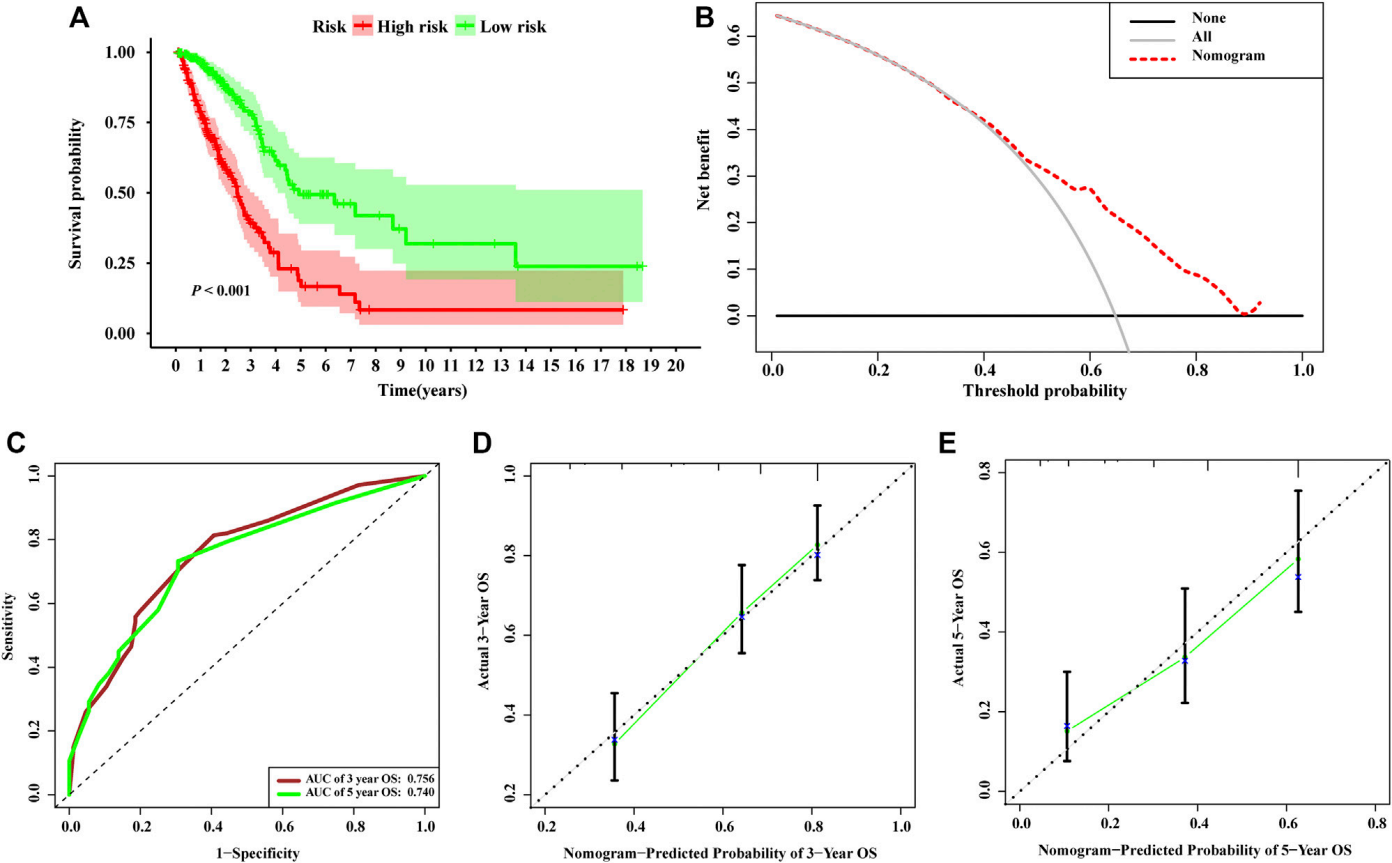
Assessment of the nomogram incorporating the risk scores generated from the signature. **(A)** Kaplan–Meier survival analysis curves of the high-risk group and low-risk group divided by the nomogram in TCGA-LUAD set. **(B)** Decision curve analysis evaluating the clinical usefulness of the nomogram for LUAD patients **(C)** Time-dependent ROC curves at 3- and 5-year follow-up. The calibration curves show the prediction of the nomogram for 3-year **(D)** and 5-year **(E)**.

Based on the risk scores calculated by the nomogram, LUAD patients in the entire set were classified into different risk groups by the median value of the risk score. Figure 6A shows that low-risk group patients possessed significantly better prognoses than those in the high-risk group (*p* < 0.001). The result of DCA demonstrated that majority of the red dashed curve was in the area above the gray and the black solid lines, illustrating a higher net benefits could be acquired by using the nomogram to make decision (Figure 6B). In addition, the ROC analyses showed satisfactory discrimination performance of the model with an AUC of 0.756, and 0.740 at 3- and 5-year follow-up (Figure 6C). Further, a good agreement between the nomogram prediction and actual observation was illustrated *via* calibration curves (Figures 6D,E). Consequently, promising predictive value was revealed for this prognostic integrated nomogram.

### Functional Enrichment Analysis and Immune Infiltration Analysis

**FIGURE 7:**
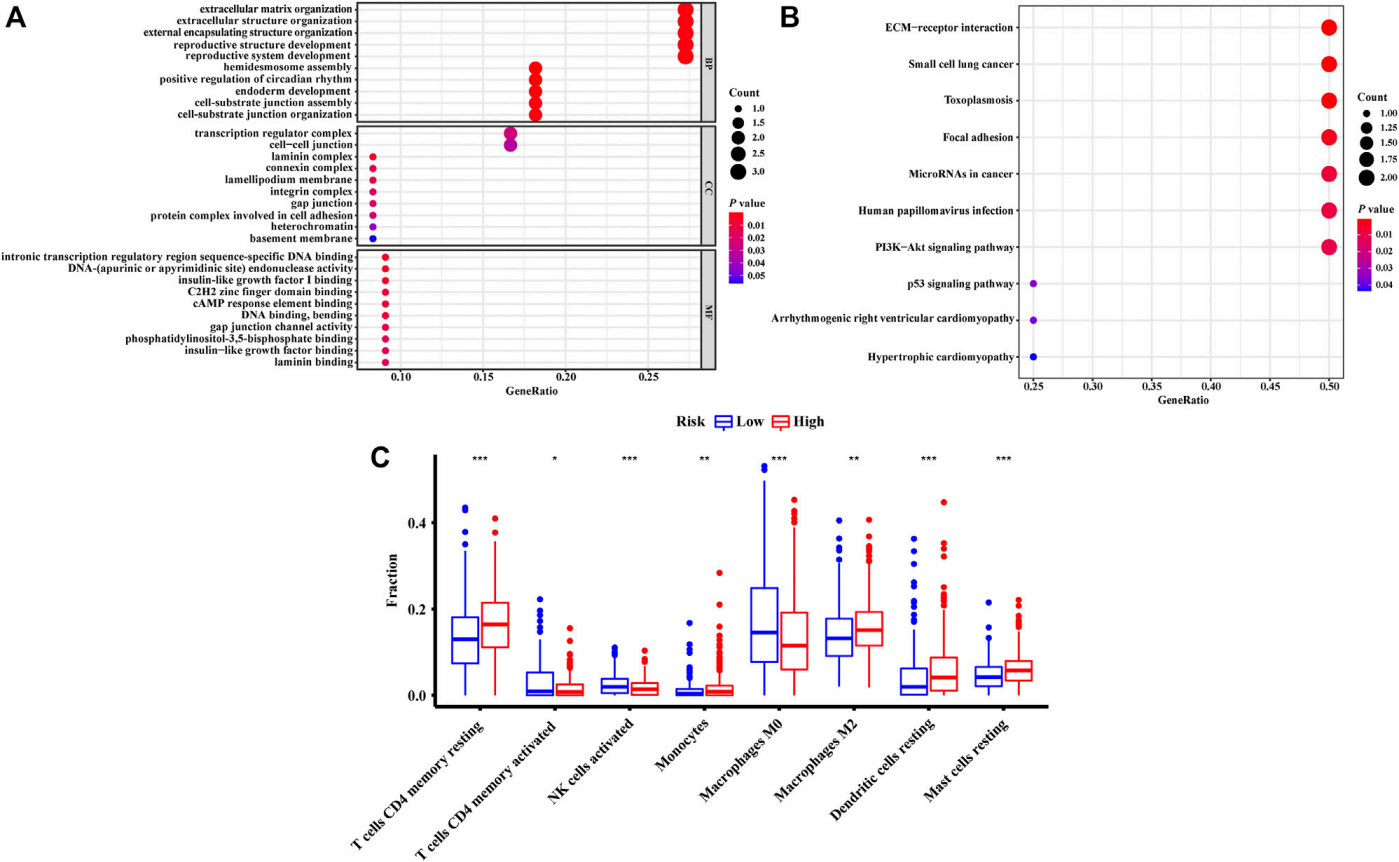
Functional enrichment analysis and the characteristics of immune infiltration of different groups defined by the signature. The GO terms **(A)** and KEGG terms **(B)** enriched by the co-expressed genes related to necroptosis-related lncRNA. **(C)** Differences in abundance of tumor-infiltrating immune cells among two risk groups stratified by the necroptosis-related lncRNA signature.

To investigate the potential biological functions and the immune infiltration status associated with NRLRs, we performed co-expression analysis to screen out the NRLRs-related protein-coding genes. Only LINC00941 has been investigated in previous studies (Wang et al., 2019; Wu et al., 2021). Therefore, the functional enrichment analysis focused on this necroptosis-related lncRNA, LINC00941. |Pearson correlation coefficients| > 0.5 and *p* < 0.001 as the criteria selected 12 protein-coding genes from the RNA transcriptome data of the TCGA-LUAD. Among these protein-coding genes, the expression of these genes was positively associated with the expression of LINC00941, except for TMEM125 and NKX2-1 (Supplementary Figure S3A). As shown in Figure 7A, the GO functional enrichment analysis demonstrated that the correlated genes were mainly clustered in several biological processes or molecular functions such as extracellular matrix organization, extracellular structure organization, transcription regulator complex, and intronic transcription regulatory region sequence-specific DNA binding. At the same time, KEGG terms of correlated genes were significantly enriched in several signaling pathway such as extracellular matrix-receptor (ECM-receptor) interaction, toxoplasmosis, and focal adhesion pathway (Figure 7B). Altogether, these analyses suggested that the NRLRs-related protein-coding genes may be mainly correlated with tumor migration and metastasis in LUAD.

The abundances of tumor-infiltrating immune cells were estimated by TIMER and CIBERSORT algorithms. The results generated from the TIMER algorithm demonstrated that the six kinds of immune cell infiltration were negatively correlated with the risk score calculated by the NRLSig, though only B cells and dendritic cells showed significant association with the prognosis of LUAD patients (Supplementary Figures S4A–F). Moreover, the boxplots from the CIBERSORT algorithm showed that abundances of resting memory CD4^+^ T cells, monocytes, M2 macrophages, resting dendritic cells, and resting mast cells were markedly enriched in the high-risk group compared to the low-risk group. On the contrary, the abundances of activated memory CD4^+^ T cells, activated natural killer (NK) cells, and M0 macrophages in the high-risk group were significantly lower than in the low-risk group (Figure 7C). In summary, the association between the risk scores generated from the NRLSig and tumor-infiltrating immune cells were assessed, and the results demonstrated that the risk level of LUAD patients was related to the distribution difference of immune infiltration cells.

### Validation of Necroptosis-Related lncRNAs Expression in Cell Lines and Tissue Samples

**FIGURE 8:**
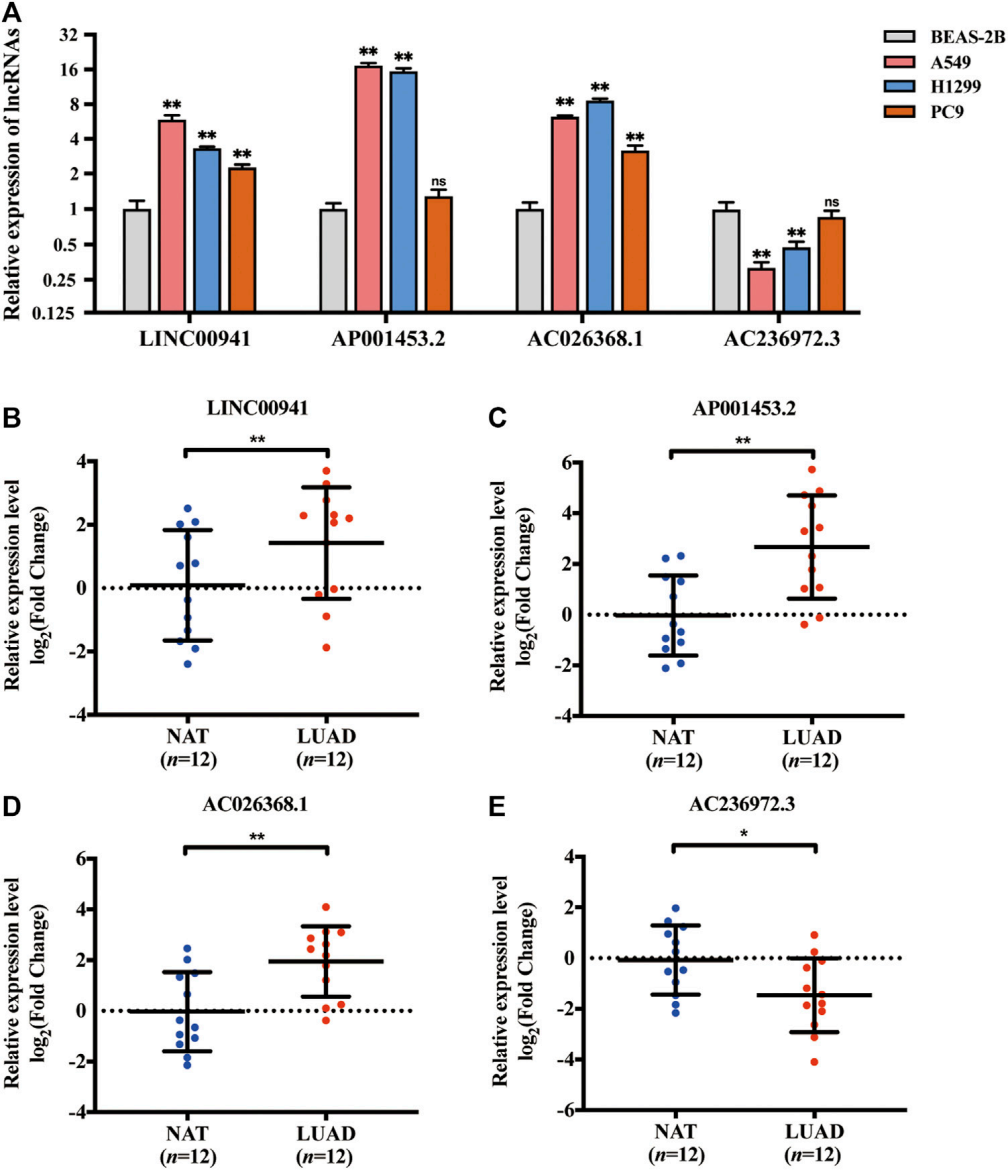
Validation of the expression of the selected necroptosis-related lncRNA in cell lines and tissues. **(A)** The relative expression of 4 necroptosis-related lncRNAs in lung adenocarcinoma cell lines (A549, H1299, and PC9) with human bronchial epithelial cell line (BEAS-2B). **(B–E)** The relative expression of LINC00941, AP001453.2, AC026368.1, and AC236972.3 in 12 pairs of lung adenocarcinoma tissue samples. *p < 0.05; **p < 0.01.

The expression levels of selected NRLRs were further evaluated and validated in cell lines and tissues. As illustrated in Figure 8A, the expression levels of LINC00941, AP001453.2, and AC026368.1 were significantly higher in LUAD cell lines, including A549, H1299, and PC9, than those in human normal lung epithelial cells (BEAS-2B), while AC236972.3 exhibited the opposite trend. We also evaluated the expression level of these 4 lncRNAs in 12 pairs of LUAD tissues and NAT. Consistent expression trends were observed in these tissue samples. LINC00941, AP001453.2, and AC026368.1 showed higher expression levels in LUAD tissues than in NAT, but the expression of AC236972.3 was significantly lower in tumor tissues than in NAT (Figures 8B–E). These results further confirmed the correctness of the above bioinformatics analyses.

## Discussion

In recent years, with the development of next-generation sequencing, accumulating non-coding RNAs and protein-coding genes have been identified as prognostic predictor for cancer patients (Borad et al., 2016; Lagana et al., 2016; Li N. et al., 2020). In current clinical practice, the traditional staging system may not be optimal for individualized prognostic prediction for LUAD patients (Yao et al., 2021). Thus, it is urgently needed to investigate biomarkers related to tumor diagnosis and prognosis. LncRNAs, a kind of non-protein-coding RNAs, are widely expressed in different tissues and participate in various kinds of biological processes in malignant tumors. Necroptosis, a novel form of regulated cell death, possesses a mechanistic resemblance to apoptosis and a morphological resemblance to necrosis. Several potential lncRNAs have been identified as the regulators for necroptosis. Therefore, NRLRs have also attracted plenty of attention for promising prognostic value in LUAD.

To the best of our knowledge, this is the first study to identify and comprehensively analyze prognostic NRLRs in LUAD. A signature based on 4 NRLRs and a predictive model incorporating this signature were developed in the present study, and this nomogram showed higher discriminatory accuracy for predicting OS of LUAD patients compared to models constructed in previous studies (Liu and Yang, 2021; Yao et al., 2021). Additionally, we also investigate the enriched biological functions and immune infiltration status related to NRLRs in LUAD cohort.

According to the Sankey diagram, we identified 4 lncRNAs that were related to 25 NRGs. Among these NRGs, dynamin 1-like (DNM1L), a regulator of necroptosis by activating mitochondrial fission, was correlated with LINC00941 and AC026368.1 (Remijsen et al., 2014). It also suggests a potential pivotal role in tumorigenesis and progression of NSCLC (Furukawa et al., 2005). Peptidylprolyl isomerase A (PPIA), associated with AP001453.2, is an intracellular protein released early in the process of necroptosis and has been identified to be a biomarker for this form of cell death (Cabello et al., 2021). Sirtuin 3 (SIRT3), which is related to AC236972.3, can inhibit the proliferation of human small-cell lung cancer cells by promoting apoptosis and necroptosis (Tang X. et al., 2020). Moreover, LINC00941 was reported that its overexpression could accelerate tumor progression in NSCLC *via* miR-877-3p/VEGFA axis (Ren et al., 2021). The biological function or mechanism of other lncRNAs are still unclear. The understanding of these newly identified lncRNAs needs further mechanistic study.

To explore the potential functions or mechanisms of the lncRNAs in NRLSig, co-expression network analysis and functional enrichment analysis were conducted. The GO enrichment analysis illustrated that the co-expressed genes significantly enriched in several functions. First, a large amount of co-expressed genes was associated with organization of extracellular matrix (ECM). Genetic and epigenetic changes in lung cancer may lead to the conversion of ECM, such as misexpression of collagens, proteases and integrins in the tumor microenvironment, which could consequently cause tumor progression (Götte and Kovalszky, 2018; Paolillo and Schinelli, 2019). In addition, NK2 homeobox 1 (NKX2-1) and aryl hydrocarbon receptor nuclear translocator-like (ARNTL2) both act as a transcription regulator. Loss of NKX2-1 could lead to the recruitment of tumor-associated neutrophils which promote the proliferation of lung squamous cell (Mollaoglu et al., 2018), and high expression of ARNTL2, which could drive metastatic self-sufficiency and predict poor prognosis for LUAD patients (Brady et al., 2016). Moreover, integrin subunit alpha 6 (ITGA6) could regulate lung differentiation in stress response by mediating cell adhesions to laminin (Sanchez-Esteban et al., 2006). Furthermore, several KEGG terms were also enriched. Laminin subunit gamma 2 (LAMC2), enriched in most of the KEGG signaling pathways in present study, was found to promote tumor proliferation, metastasis, and vascular regeneration through ECM-receptor interaction and focal adhesion (Wang et al., 2020).

We found that the abundances of activated memory CD4^+^ T cells and activated NK cells were significantly lower in the high-risk group compared to the low-risk group, while M2 macrophages were enriched in the high-risk group. This phenomenon suggested that the high-risk group patients may possess deteriorated immune status and immune function. A previous study revealed that interleukin 12 (IL-12) could promote the proliferation and tumor suppression of memory CD4^+^ T cells presenting in the tumor microenvironment (TME) of lung cancer (Broderick et al., 2005). NK cells, an effecter lymphocyte of the innate immune system, could control tumor proliferation and metastatic spread (Sivori et al., 2021). Further, since M2 macrophages could secrete a series of anti-inflammatory molecules to function as pro-tumoral factors, high abundance of tumor-infiltrating M2 macrophages was associated with unfavorable prognosis of NSCLC patients (Jackute et al., 2018). In general, the dysregulation of the immune status of TME may lead to a discrepancy in survival prognosis among the high-risk group and low-risk group stratified by the prognostic NRLSig.

Several limitations in our study still need to be considered, though we applied many methods to adjust and validate our signature. First, as this was a retrospective study based on public databases, some information related to lung cancer may be unavailable, such as smoking status. Second, we used a single data source in this study. Though we applied internal validation to test our findings, whether the performance of the model in an external cohort would be similarly satisfactory still require further validation. In this study, the RNA expression data and survival information of 163 LUAD patients had been retrieved from the GSE3141 series and GSE37745 series matrices from Gene Expression Omnibus (GEO) database (https://www.ncbi.nlm.nih.gov/geo/). However, we could not acquire sufficient information of lncRNAs in the GEO cohort because the scale of commercial sequencing data from the GEO dataset was much smaller compared to the size of RNA sequencing data from the TCGA dataset. Third, the *in vitro* and *in vivo* experiments will be required to further elucidate the biological mechanism or prognostic value of NRLRs in LUAD.

In conclusion, we proposed a signature, constructed based on 4 lncRNAs biomarkers, that could independently predict the prognosis of LUAD patients. Moreover, the possible biological functions and immune status of the 4 NRLRs could provide novel insights for further research on the molecular mechanisms of tumorigenesis and progression of LUAD. In all, the NRLRs identified in this study may offer promising therapeutic targets or prognostic predictors for LUAD patients.

## Data Availability

Publicly available datasets were analyzed in this study. This data can be found here: TCGA-LUAD, The Cancer Genome Altas (https://portal.gdc.cancer.gov).
