# Supplementary material for: Construction of a Novel Prognostic Signature in Lung Adenocarcinoma Based on Necroptosis-Related lncRNAs
Source: Front Genet. 2022 Jul 22;13:833362. doi: 10.3389/fgene.2022.833362 (PMC9354127; doi:10.3389/fgene.2022.833362)
Supplement: Supplementary file 2 [file Table1.DOCX]

**Table S1. Necroptosis-related genes**

TNF

TNFRSF1A

TRADD

TRAF2

TRAF5

RIPK1

BIRC2

BIRC3

XIAP

RBCK1

RNF31

SHARPIN

SPATA2L

SPATA2

CYLD

FADD

CASP8

CFLAR

RIPK3

CYBB

CAMK2A

CAMK2D

CAMK2B

CAMK2G

SLC25A4

SLC25A5

SLC25A6

SLC25A31

PPID

VDAC1

VDAC2

VDAC3

GLUD2

GLUD1

GLUL

PYGL

PYGM

PYGB

MAPK8

MAPK10

MAPK9

FTH1

FTL

PLA2G4E

PLA2G4A

JMJD7-PLA2G4B

PLA2G4B

PLA2G4C

PLA2G4D

PLA2G4F

ALOX15

CAPN1

CAPN2

SMPD1

MLKL

PGAM5

DNM1L

NLRP3

PYCARD

CASP1

IL1B

CHMP2A

CHMP2B

CHMP3

RNF103-CHMP3

CHMP4B

CHMP4A

CHMP4C

CHMP6

VPS4B

VPS4A

CHMP1B

CHMP1A

CHMP5

CHMP7

TRPM7

IL1A

IL33

HMGB1

TNFSF10

TNFRSF10A

TNFRSF10B

FASLG

FAS

FAF1

IFNA1

IFNA2

IFNA4

IFNA5

IFNA6

IFNA7

IFNA8

IFNA10

IFNA13

IFNA14

IFNA16

IFNA17

IFNA21

IFNB1

IFNG

IFNAR1

IFNAR2

IFNGR1

IFNGR2

JAK1

JAK2

JAK3

TYK2

STAT1

STAT2

STAT3

STAT4

STAT5A

STAT5B

STAT6

IRF9

EIF2AK2

TLR4

TICAM2

TICAM1

TLR3

ZBP1

USP21

SQSTM1

HSP90AA1

HSP90AB1

TNFAIP3

PARP1

BID

BAX

AIFM1

H2AX

H2AC20

H2AC12

H2AC1

H2AW

H2AB3

H2AC8

H2AC4

MACROH2A2

MACROH2A1

H2AC19

H2AJ

H2AB1

H2AC17

H2AC18

H2AC11

H2AC21

H2AZ2

H2AC7

H2AZ1

H2AC15

H2AC6

H2AC13

H2AC14

H2AC16

H2AB2

PPIA

BCL2

TSC1

TRIM11

IPMK

ITPK1

SIRT3

MYC

TNFRSF1B

PANX1

OTULIN

USP22

MAP3K7

DIABLO

DNMT1

BRAF

AXL

ID1

CDKN2A

HSPA4

STUB1

FLT3

HAT1

SIRT2

SIRT1

PLK1

MPG

BACH2

GATA3

MYCN

ALK

ATRX

TERT

SLC39A7

IDH1

IDH2

KLF9

HDAC9

LEF1

BNIP3

CD40

BCL2L11

EGFR

DDX58

TARDBP

APP

TNFRSF21
