# Supplementary figures and images for: Construction of a Novel Prognostic Signature in Lung Adenocarcinoma Based on Necroptosis-Related lncRNAs
Source: Front Genet. 2022 Jul 22;13:833362. doi: 10.3389/fgene.2022.833362 (PMC9354127; doi:10.3389/fgene.2022.833362)

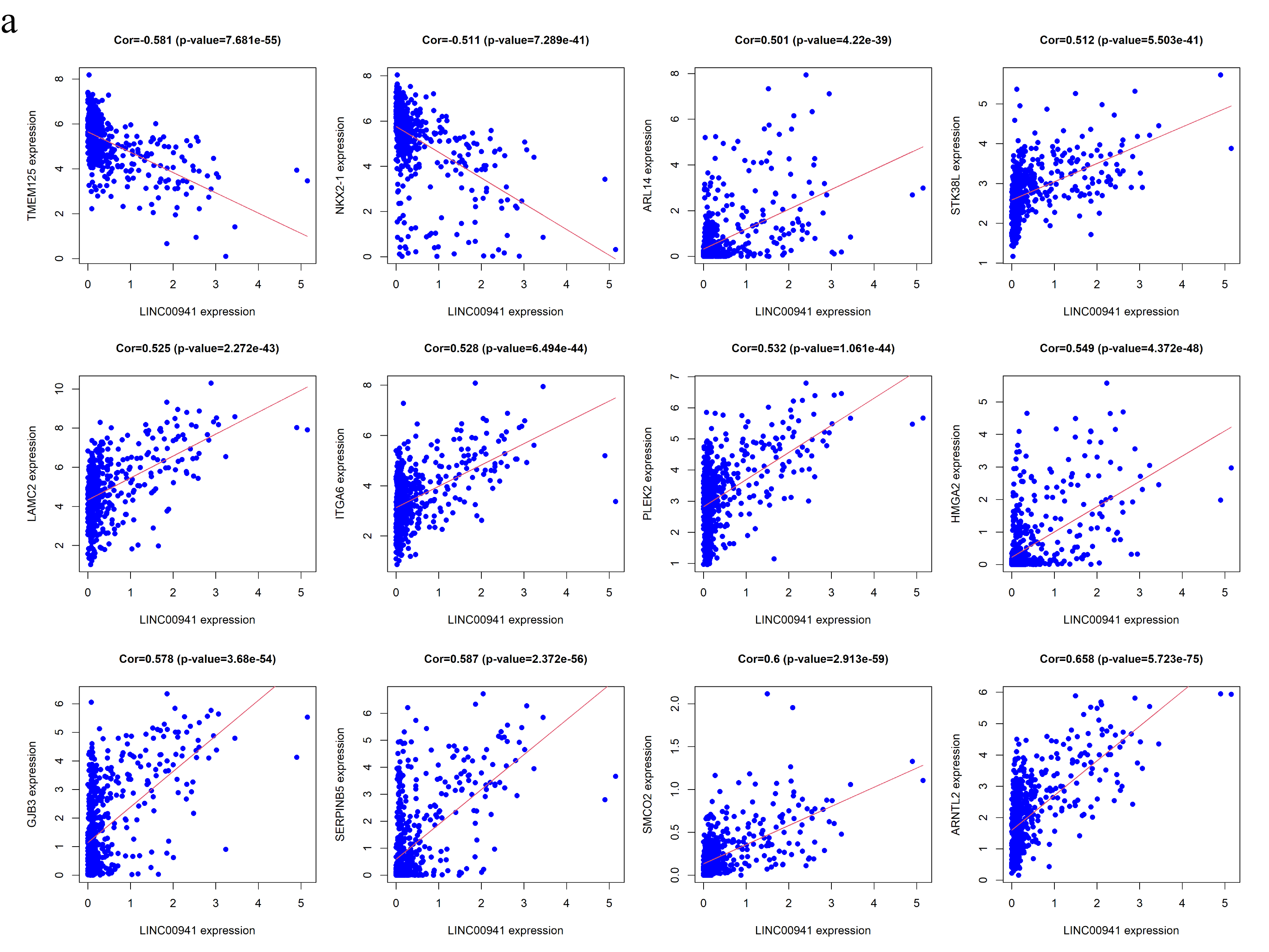

Supplement: Supplementary file 3 [file Image3.TIF]

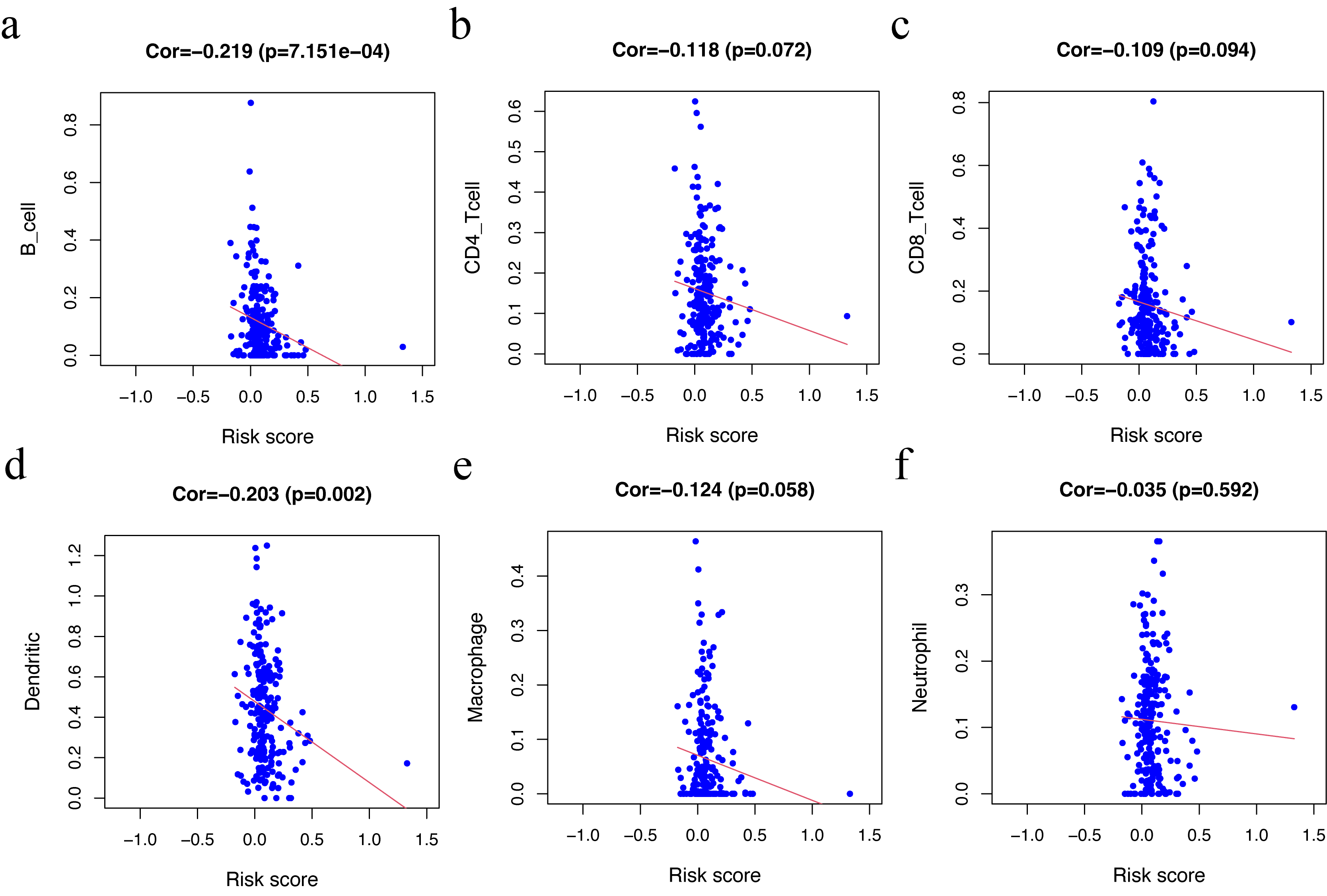

Supplement: Supplementary file 4 [file Image4.TIF]

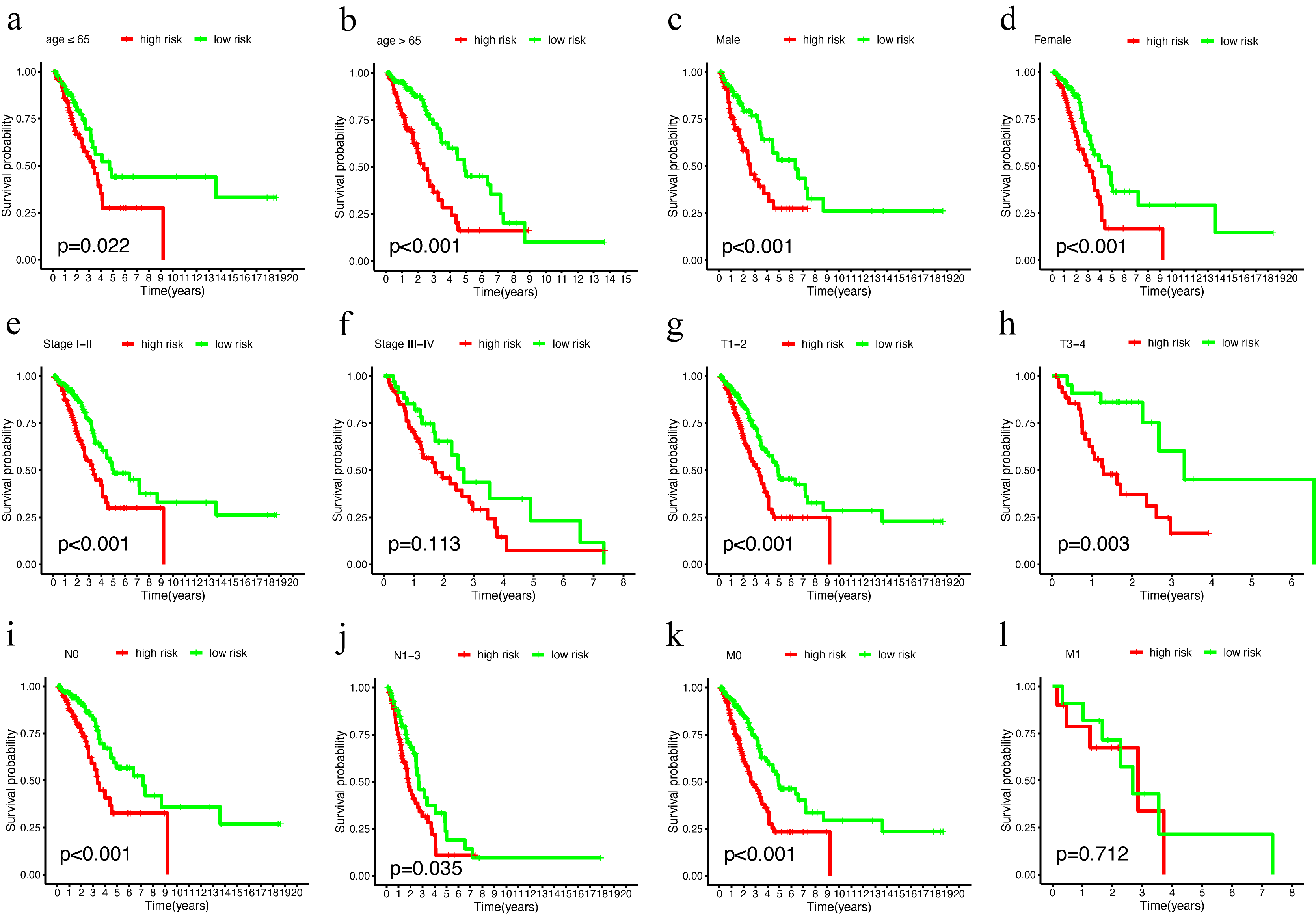

Supplement: Supplementary file 5 [file Image2.TIF]

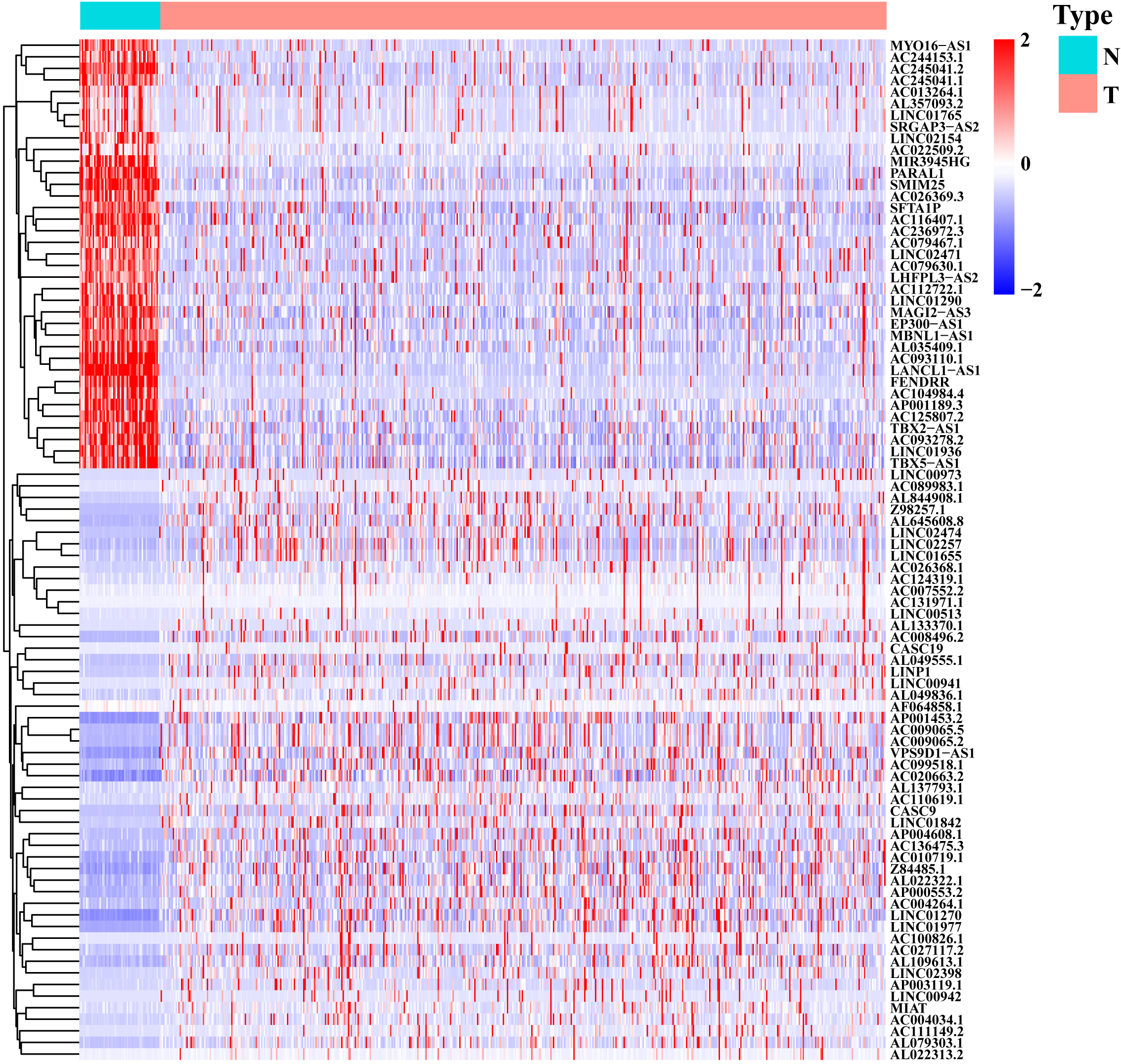

Supplement: Supplementary file 6 [file Image1.TIF]
