# Supplementary material for: Construction of a Novel Prognostic Signature in Lung Adenocarcinoma Based on Necroptosis-Related lncRNAs
Source: Front Genet. 2022 Jul 22;13:833362. doi: 10.3389/fgene.2022.833362 (PMC9354127; doi:10.3389/fgene.2022.833362)
Supplement: Supplementary file 7 [file Table3.DOCX]

**Supplementary captions**

**Figure S1. The heatmap of the selected 88 necroptosis-related lncRNA showing the expression level of each lncRNA in** **the TCGA cohort.**

**Figure S2. Survival analyses for the** **necroptosis-related lncRNA signature in subgroups.** Kaplan–Meier survival curves of overall survival in subgroups stratified by age **(a, b)**, gender **(c, d)**, stage **(e, f)**, T stage **(g, h)**, N stage **(i, j)**, or M stage **(k, l)** between the low-risk group and high-risk group.

**Figure S3. Protein-coding genes co-expressed with the necroptosis-related lncRNA. (a)** The correlation between the necroptosis-related lncRNA and 12 protein-coding genes.

**Figure S4. Association between immune cell infiltration and the risk scores** **generated by the signature.** The correlation between the risk scores and the infiltration of B cell (a), CD4+ T cell (b), CD8+ T cell (c), dendritic cell (d), macrophage (e), and neutrophil (f).
